# Household costs and time to seek care for pregnancy related complications: The role of results-based financing

**DOI:** 10.1371/journal.pone.0182326

**Published:** 2017-09-21

**Authors:** Jobiba Chinkhumba, Manuela De Allegri, Jacob Mazalale, Stephan Brenner, Don Mathanga, Adamson S. Muula, Bjarne Robberstad

**Affiliations:** 1 Department of Public Health, University of Malawi, College of Medicine, School of Public Health and Family Medicine, Blantyre, Malawi; 2 Center for international Health. University of Bergen, Bergen, Norway; 3 Institute of Public Health, University of Heidelberg, Heidelberg, Germany; Population Council, KENYA

## Abstract

Results-based financing (RBF) schemes–including performance based financing (PBF) and conditional cash transfers (CCT)-are increasingly being used to encourage use and improve quality of institutional health care for pregnant women in order to reduce maternal and neonatal mortality in low-income countries. While there is emerging evidence that RBF can increase service use and quality, little is known on the impact of RBF on costs and time to seek care for obstetric complications, although the two represent important dimensions of access. We conducted this study to fill the existing gap in knowledge by investigating the impact of RBF (PBF+CCT) on household costs and time to seek care for obstetric complications in four districts in Malawi. The analysis included data on 2,219 women with obstetric complications from three waves of a population-based survey conducted at baseline in 2013 and repeated in 2014(midline) and 2015(endline). Using a before and after approach with controls, we applied generalized linear models to study the association between RBF and household costs and time to seek care. Results indicated that receipt of RBF was associated with a significant reduction in the expected mean time to seek care for women experiencing an obstetric complication. Relative to non-RBF, time to seek care in RBF areas decreased by 27.3% (95%CI: 28.4–25.9) at midline and 34.2% (95%CI: 37.8–30.4) at endline. No substantial change in household costs was observed. We conclude that the reduced time to seek care is a manifestation of RBF induced quality improvements, prompting faster decisions on care seeking at household level. Our results suggest RBF may contribute to timely emergency care seeking and thus ultimately reduce maternal and neonatal mortality in beneficiary populations.

## Introduction

Approximately 4% of pregnant women in sub-Saharan Africa suffer from severe obstetric complications during the course of their pregnancy[1], while delayed effective management is recognised as a major determinant of maternal mortality in low-income settings[2]. Thaddeus and Maine have posited that delays in seeking obstetric emergency care occur at three levels. The first delay involves making decisions to seek care and occurs at household level once an emergency arises. Gender and power dynamics within households, perceptions of symptom severity, quality of services available and the financial costs of care influence decision making process at this level. The second delay relates to transport to a health facility once a decision to seek emergency care is made and occurs at community level. Availability and affordability of transport are important community level factors. The third delay relates to obtaining timely emergency care after a woman with an obstetric emergency presents to health workers and occurs at health facility level. At this level, facility readiness and provider skills are critical in providing definitive care[3].

At facility level, a range of effective interventions to manage obstetric complications is available in resource poor settings, including management of hemorrhage, eclampsia, sepsis and obstructed labor [4–6]. Still, in resource poor countries, many pregnant women either underutilize obstetric emergency services or present too late at health facilities in case of need[7, 8].

Analysis of recent Demographic and Health Surveys (DHS) has identified lack of money as an important obstacle to maternal services use[9]. To enhance utilization of maternal services, some governments in low and middle income countries are experimenting with results-based financing (RBF) strategies, including both demand-side(e.g. conditional cash transfers for clients) and supply-side (e.g. performance based financing for workers) [10–14].

The mechanisms through which demand-side and supply-side incentives are expected to operate have been outlined in the literature. By making cash transfers conditional on health care seeking, the financial rewards can be used to shape household behaviour leading to increased use of maternal health services. Conditioning acts through the price effect mechanism: a “price” is incurred (loss of a financial reward) if a particular behaviour is not performed[15]. Alternatively, cash transfers, if large or frequent enough, can increase household incomes. Increased income is believed to improve the ability of poor households to overcome economic barriers, leading to increased expenditures on normal goods e.g. healthcare. In this aspect, the cash transfers are anticipated to change consumption behavior through income effect as predicted by microeconomic theory[16]

Through the provision of performance incentives, health facilities and health workers receive payments based on the achievement of a set of pre-defined quantity and quality targets[10]. Building on the constructs of principal-agent theory, financial incentives are expected to redirect health workers’ behavior towards provision of better quality care and to attract more patients to health facilities[13].

While there is emerging evidence on the impact of RBF on service use and quality of service delivery[12–14], little is known on the RBF impact on household costs or time to seek care, especially for women experiencing obstetric complications. Cost studies on obstetric complications commonly focus on describing the high economic burden of individual maternal morbidities[17], or the differences in costs between women surviving or dying from complications[18] or the cost differentials in women with and without complications[19]. Similarly, although perceived and actual costs of care are linked to delays in seeking emergency care, little is known about the association between RBF and timeliness for emergency obstetric care[20]. Filling this knowledge gap is important as it may offer an understanding if and how the provision of financial incentives has potential to affect household costs and reduce time to seek care for women experiencing obstetric complications.

Our study aimed to fill this gap in knowledge through an analysis directed specifically at identifying the impact of RBF on i) household costs and ii) time to seek care for women with pregnancy related complications. We focused on delays encountered at household and community levels: it was outside the scope of our study to consider delays at the facility. Our study was conducted within the framework of a larger impact evaluation related to the implementation of the RBF for Maternal and Neonatal Health (RBF4MNH) initiative in Malawi [21].

## Methods

### Study setting

Malawi is a low income country with a population of 17 million. Its gross domestic product (GDP) is 4.3 billion US$, of which 4.2% is spent on healthcare[22]. Total expenditure on reproductive health (RH) rose from US$50.1 million in 2009/10 to US$74.3 million in 2010/2011, and then declined to US$63.6 million in 2011/12 [23]. On average, US$9.9 per annum was spent on RH over the same period on each woman of reproductive age (15–49 years). Routine and emergency obstetric care are provided free at all public health facilities and at selected private not for profit facilities contracted by the government through Service Levels Agreements. Although Malawi imposes no formal fees for emergency obstetric care (EmOC), evidence shows that some medical costs are still shifted towards patients due to stock outs of drugs and other items needed for surgery[24]. In each district, health centers provide basic emergency obstetric care (BEmOC) and refer complicated cases to respective district hospitals, which provide comprehensive emergency obstetric care (CEmOC). Despite the government’s financial commitment towards reproductive health, maternal mortality ratio in the country is still high at 574/100,000 live births[25].

### The results-based financing strategy

Details of the RBF4MNH initiative are provided elsewhere [21]. Briefly, the aim of RBF4MNH initiative is to reduce maternal and neonatal mortality through increased access and improved quality of service delivery. The RBF4MNH initiative includes both supply-side and demand-side conditional financial incentives. Supply-side incentives are paid on a quarterly basis to health facilities upon attainment of pre-agreed performance targets: 70% to be divided as top-up among health workers providing maternal and child health (MCH) services and 30% to be invested in improving facility infrastructure and supplies. Hospitals receive 60% for top-up and 40% for investments. The demand-side incentives are paid to pregnant women,irrespective of income or socio-economic status, residing in the designated health facility catchment areas upon delivering in the designated health facility or if referred at the district hospital. The cash transfers, averaging US$10.50 per woman[26], consist of a flat lump sum (US$ 4.0) and of a variable portion, depending on whether a woman remains at a facility 48 hours postpartum and on the distance travelled to access facility care. Although the transfer is only disbursed at delivery, women must register already while pregnant during antenatal visit. Health surveillance agents are responsible to verify women’s village of residence to confirm their eligibility and determine the amount to be received. To ensure sufficient capacity for obstetric care service provision, the RBF4MNH initiative was preceded by a one-time infrastructural and supply upgrade. Additional facility investments were considered to occur based on the earmarked performance rewards. The Ministry of Health (MoH), through district health management teams (DHMTs), is the lead agent in the RBF4MNH implementation. Technical assistance is provided by Options Consulting Services Limited.

### Study sites and RBF implementation design

**Fig 1:**
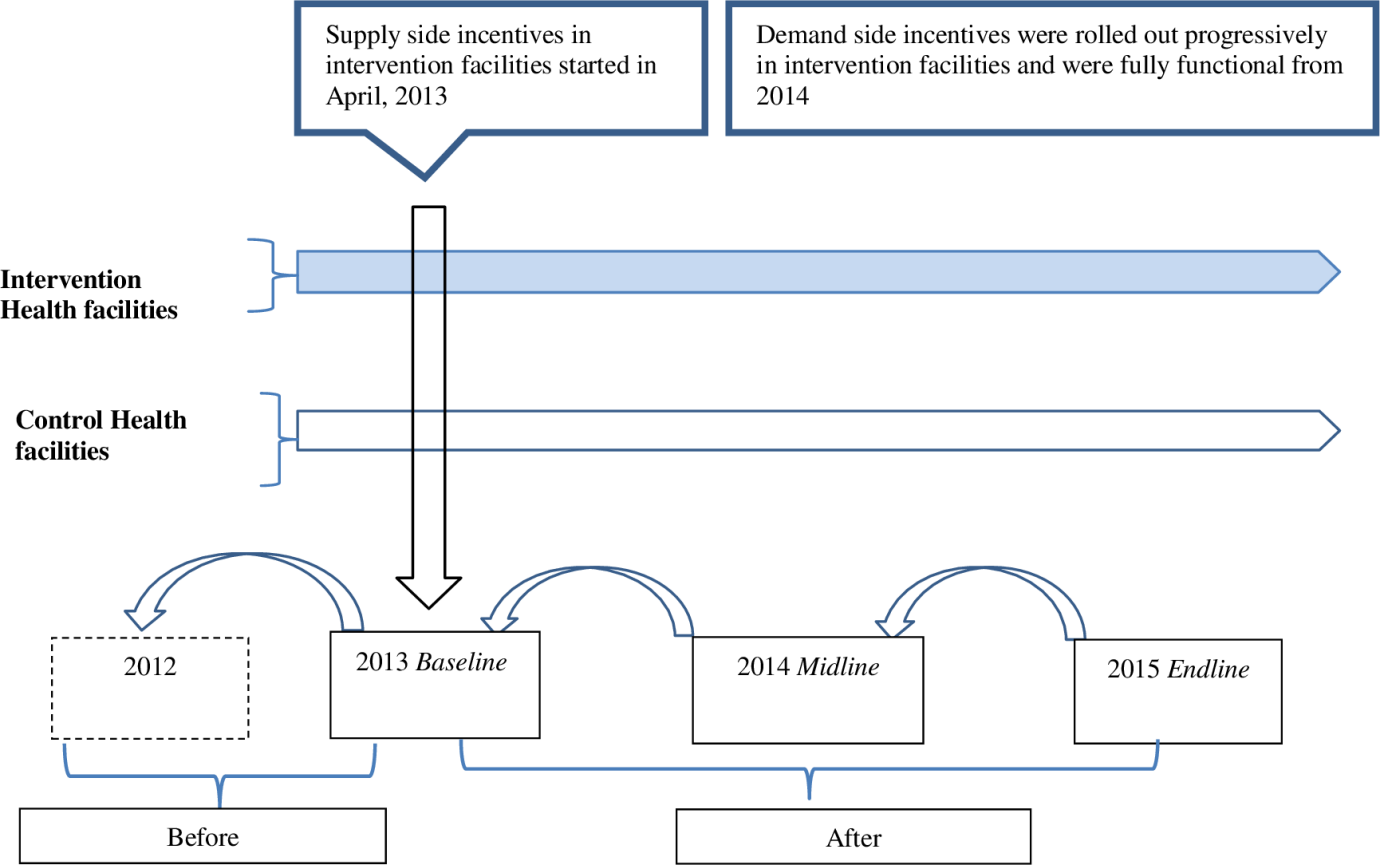
Provides information on incentives and data collection periods for evaluation of the Malawi RBF4MNH initiative. The vertical arrow indicates when supply-side incentives to health workers were applied to Intervention facilities. The intervention facilities in addition received demand-side incentives for women which were fully functional from 2014. Blue horizontal arrow represents interventin facilities. White horozontal arrow represents control facilities. Horizontal axis shows the before and after periods and timing of data collection. Back pointing arrows indicate the 12 months recall period data was collected during each survey round.

In 2013, the Malawi MoH selected four districts, Balaka, Dedza,Ntcheu and Mchinji with a combined population of 1.9 million to pilot the RBF4MNH initiative[21]. The districts were purposefully selected so that they were relatively representative of the rest of the 28 districts in the country in terms of maternal/childhood illness patterns and administrative arrangements. Across the four districts, the MoH identified a total of 33 public health facilities (28 BEmOC and 5 CEmOC) eligible to provide EmOC services and selected 17 of those (4 district hospitals/CEmOCs and 13 BEmOCs) to be recipients of the RBF4MNH initiative. One year later, the intervention was expanded with one private not for profit mission hospital/CEmOC and 10 BEmOCs (including 5 private not for profit facilities). The supply-side component was rolled out at the selected CEmOC and BEmOC facilities soon after the official launch of the program in April 2013. Due to implementation challenges, the demand-side component became fully effective across facilities only one year later. **Fig 1** illustrates how in terms of intervention exposure, this arrangement entailed that women needing complication care residing in the catchment areas of RBF4MNH facilities (hereafter defined as RBF group) were likely affected by the supply-side incentives provided at both BEmOC and CEmOC level as well as by the conditional cash transfers, while women residing in the catchment areas of non-RBF4MNH facilities (hereafter defined as non-RBF group) were likely affected by the supply-side incentives only if referred to seek complication care in a CEmOC facility.

### Data sources

Data for this study were obtained through three repeated cross-sectional household surveys, conducted at baseline, midterm, and endpoint: April to May 2013, June to July 2014 and June to July 2015 respectively (S1 Data). The data were collected as part of a larger study evaluating the impact of the RBF4MNH strategy, the detailed data collection design and procedures have already been published [27]. Before the first survey, enumeration areas were randomly selected for each health facility. Within each enumeration area, eligible women were interviewed. The surveys targeted all women having completed a pregnancy in the 12 months prior to the survey date. The analysis presented here is limited to the truncated sample of women who reported having experienced a pregnancy-related complication at any point in the course of their pregnancy. **Fig 1** provides details on timing of data collection and appropriate recall periods.

Trained interviewers collected data from the women using a structured questionnaire, programmed digitally and administered using tablet computers. The questionnaire was administered in Chichewa, the common local language. Information was collected on the women’s social demographic features, self-reported complications and hospital admissions due to complications related to the pregnancy completed within the prior 12 months. The information on self-reported complications was collected in the form of lay person descriptions of a combination of symptoms and signs suggestive of common obstetric complications[20]. This information was validated by interviewers using formal diagnosis recorded in the women’s health passports, where possible. For each self-reported complication, information about relevant out-of-pocket expenditures on medical costs (consultations, drugs, surgical procedures, radiological and laboratory fees) transport costs, food and accommodation were recorded. Time use for seeking and obtaining care for both patients and their informal caregivers was also recorded. All women reporting a complication were asked if they sought care. If the response was yes, the women were then asked to report how quickly after symptoms onset they had decided to seek care, and how many days elapsed before they presented to a facility once decision to seek care was made.

Ethical approval for the study was obtained from University of Malawi, College of Medicine Research and Ethics Committee (COMREC) protocol P.02/13/1353 and Ethics Committee of Faculty of Medicine of the University of Heidelberg, Germany, protocol number S-256/2012. Permission to conduct the study was sought from district and village authorities. Written informed consent was obtained from all women prior to the interview.

### Variables definitions and measurements

#### Dependent variables

In line with the two objectives, we defined two dependent variables a) Total costs and b) Time to seek care. Total costs were defined as the sum of both direct costs (e.g. medical and transport fees) and indirect costs incurred for each reported complication. We estimated costs of time taken to seek care and actually spent at health facilities using a simplified human capital approach[28]. For each reported complication, we quantified and added up lost patient and informal caregiver’s time in days. Given the high level of self or informal employment in our sample (>80%) and the lack of job specific mean wage information for those in formal employment, we used minimum wages to value lost productivity for both the formally and informally employed. While using minimum wage for those formally employed would bias their wages downwards, this would be offset by using minimum wage for the majority of self or informally employed who probably earn less than the minimum wage. Productivity loss (opportunity cost) was estimated as the product of the time lost and daily minimum wage pertaining to the survey year. Reported minimum wages per day in US$ were 0.87, 1.30, 1.25 for years 2013,2014 and 2015 respectively [29].To compare the costs reported over the years, we used annual Consumer Price Index (CPI) increases from 2013 to 2014, 2014 to 2015 and 2014 to 2015 respectively to adjust the 2013(baseline) and 2014 (midline) costs to 2015(endline) values (1US$ = 550MK). Hereafter, we refer to total costs simply as costs, unless stated otherwise. Time to seek care was defined as duration in days a woman with a reported complication took to present for care at a health facility after symptoms onset. Hereafter, we refer to time to seek care simply as time.

#### Independent variables

The main exposure was receipt of RBF (PBF + CCT) for women in designated health facility areas. To control for confounding in the estimation of the effect, we included independent variables identified as important determinants of care seeking[30] and that have local context and cultural relevance within the framework of understanding obstetric complications care seeking[31, 32]. The variables include age, parity, education, socio-economic status(SES), area of residence, facility type and distance to facility. We additionally included variables indicating if women were registered to receive financial incentives and for those who sought care, whether they were treated as in-patients (a proxy for disease severity) and days spent in facility. We assumed these variables would have bearing on costs. Following standard approaches[33], we generated a wealth index based on household assets ownership using principal component analysis. We used the wealth index to rank the women into three SES terciles. **Table 1** provides details of the independent variables.

**Table 1:** Independent variables definitions and measurements.

*Independent variable*	*Definition*, *measurement and coding*
Age	Continuous variable, measured in years
Parity	Number of term deliveries, categorized as 0 if< 2 term deliveries and 1 if ≥ 2 term deliveries
Married	Marital status, categorized as 0 if woman not married and 1 if woman married
Head Household	Household head status, categorized as 0 if woman did not head household and 1 if woman headed household
Educated	Primary education attainment, equivalent to 8 years of schooling, categorized as 0 if woman had no primary school leaving certificate and 1if woman had primary school leaving certificate or above
Residence	Place of residence, coded as 1 if woman stayed in an urban area and 0 otherwise.
Distance	Continuous variable measured in kilometers to the nearest formal health facility
Facility	Type of health facility, coded as 1 if a facility provided CEmOC and 0 if BEmOC.
Registered	Denotes enrollment to receive demand side incentives (CCT), coded 1 if woman registered and 0 otherwise
Inpatient	Coded as 1 if woman with self-reported complication was admitted for inpatient care and 0 otherwise
Days	Continuous variable, measured as number of nights spent in facility
Social economic status	Coded as 0 if poor, 1 if middle poor and 2 if least poor

### Conceptual framework

In settings where direct and indirect costs for obstetric complications care are substantial, the *apriori* effects of cash transfers contingent on facility delivery on household costs is not clear. It would depend on the size of the transfer, the share of women with complications (during labor/delivery) who receive cash and whether receipt of cash actually substitutes for other material support for upkeep or reduce the need for informal caregiver time. For example, if the size of the transfer is large, one would expect an increase in direct costs. If the transfers are used for upkeep while a woman is admitted for care and lessen the need for informal caregivers time/support, one might expect a decrease in indirect costs. Given this lack of clear a prediction, we attempted to answer this question empirically.

**Fig 2:**
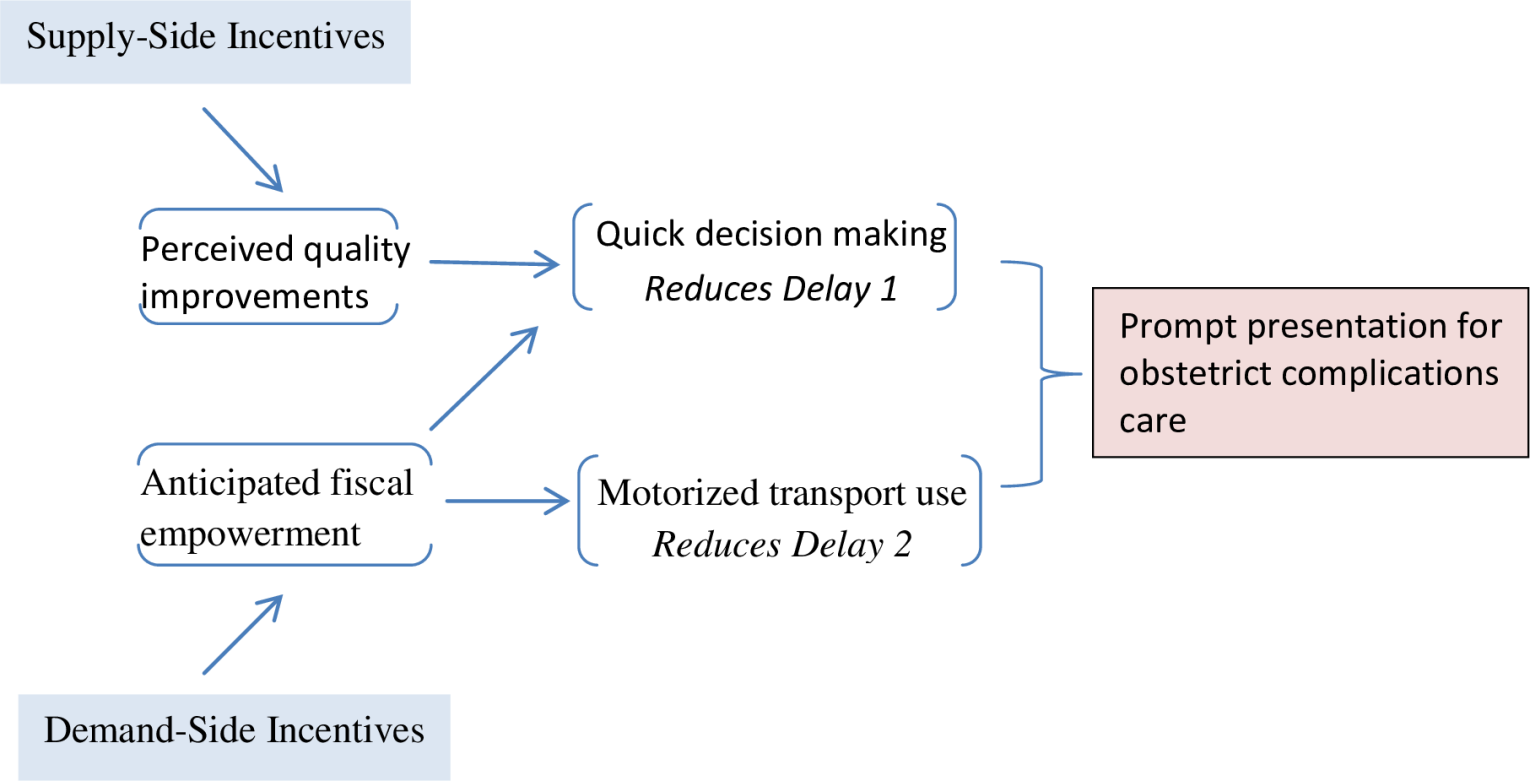
Conceptual framework depicting the association between Supply-Side Incentives, Demand-Side Incentives and prompt presentation for obstetric complications care.

Although the 3-delay model outlined by Thaddeus and Maine originally applies to delivery care, we expanded its use by applying the same set of concepts to all care pertinent to maternal care, since we assumed that the same set of barriers to access persist along the maternal care continuum. We further extended the model by linking it with performance incentives offered to health providers/facilities, cash transfers offered to women and time to seek care in **Fig 2**.The framework provides a foundation for studying the relationship between supply-side and demand-side financial incentives and time to seek care, while accounting for numerous factors that interact and may contribute to attainment of prompt emergency care.

We hypothesise that, faced with an obstetric complication, perceptions of improved quality of care at health facility resulting from supply-side incentives and guarantees of cash reimbursements would positively influence decision making at household level, leading to reduced likelihood of encountering first delay; and that promises of cash reimbursements would enable households make better transport choices(e.g. more use of motorized transport) leading to reductions in the second delay. Combined, these actions may lead to discernible reductions in average times women with obstetric complications take before presenting themselves for emergency care at health facilities.

### Statistical analysis

We provide descriptive summary statistics (means, proportions and corresponding 95% confidence intervals) for social-demographic characteristics of the women with a reported complication in the control and intervention groups. We use t-tests and chi-square tests to assess differences in means and proportions, respectively, between the two groups.

Health care data are typically positively skewed, heteroskedastic and may have nontrivial fraction of zeros making it problematic to use parametric analytic approaches[34]. To estimate populations means, E(y|x), while taking into account the non-normal distribution of health care data, generalized linear models (GLMs) are recommended as they allow for making direct inference about expected population means without recourse to complex transformations or re-transformations [35]. Given skewness of the study dependent variables (costs and time), we opted to use GLM to model the dependent variables. As total costs had trivial amounts of zeros (< 3%), a two part model, an approach often used in modeling cost data, is likely to have little effect on the overall predicted mean costs[36]. We thus limited the cost analysis into a single part prediction model.

GLMs require explicit specification of the distribution (F) of the dependent variable and the link function (g) describing how independent variables are functionally related to the dependent variable[36]. We used modified Parks test to select appropriate distribution and link functions for the study outcomes[37]. Through this test, we found that a log link with Gamma and Poisson families respectively provide best fits for the costs and time data.

The empirical GLMs took the form:

$$g(\mu_{i})=\beta_{0}+\beta_{1}{Year}_{i}{+\beta }_{2}{RBF}_{i}+\beta_{3}{RBF}_{i}*{Year}_{i}+\beta_{4}X_{I},\;y_{i}∼F$$

Where *μ*_*i*_ denotes the dependent variable of interest (costs/time) for every unit (pregnant woman seeking care for a reported complication),*Year*_i_ is a categorical variable indicating the time point taking value 0 at baseline, 1 at midline and 2 at endline, *RBF*_i,_ is an indicator variable coded 1 if the unit is in the intervention group, 0 if in the control group, X_i_ is a vector of independent factors known to influence the dependent variables as outlined above. The estimable quantities of interest are thus: β_0_, a common constant for all observations,β_1_, effect of time on each unit, β_3_ the effect of treatment (and the main target of inference) and β_4_ representing a vector of coefficients for X**(Table 1)**. Given that the decision to admit women with reported complications for in-patient care was based on clinical assessments, we considered women admitted for care a distinct subgroup. We thus ran two separate models for each of the primary study outcomes: the first model included all women with a reported complication who sought care (full model), while the second was restricted to the women who were admitted (restricted model). As the models have a log link, the exponential of coefficients should be interpreted as the ratio of arithmetic means [35]. We generated robust standard errors and corrected for clustering using the cluster command to allow for clustering of women at health facility levels. StataIC/14 (Stata-corp LP, Texas, USA) was used for the analysis.

## Results

Out of 5,622 women surveyed across the three time points: 2,219 (39.4%) reported a complication. Of these, 1,716 (30.5%) sought care and out of those, 691(12.2%) were admitted as in-patients. The women’s mean age ranged from 24.8 to 26.0 years, most (66.7–75.0%) had given birth at least twice or more and the majority (55.8–66.4%) had completed primary school education. At endline, there were significantly fewer married women (82.3 vs 89.1%), but more women heading households (13.6 vs 6.2%) in the non-RBF than the RBF group. Nearly all women in the non-RBF groups were in rural areas, a result of assigning district hospitals/CEmOCs areas into RBF. There was suggestive evidence that care seeking for reported complications was low among women in non-RBF groups at baseline and midline, but this did not reach significant levels. Care-seeking decreased substantially in both RBF and non-RBF facilities between baseline and endline. More descriptive details are shown in **Table 2**.

**Table 2:** Socio-demographic characteristics and care seeking for women with self-reported complications, by group and survey year.

	Baseline, 2013			Midline, 2014			Endline, 2015		
	*Control*	*Intervention*		*Control*	*Intervention*		*Control*	*Intervention*	
	*N = 290*	*N = 444*	*P value*	*N = 298*	*N = 351*	*P value*	*N = 198*	*N = 638*	*P value*
Age (mean)	25.6(24.9–26.4)	26.0(25.4–26.7)	0.400	25.0(24.3–25.7)	25.3(24.6–25.9)	0.606	25.1(24.2–25.9)	24.8(24.3–25.3)	0.591
Parity ≥2 (%)	75.5(70.2–80.1)	74.0(69.8–77.9)	0.666	68.4(62.9–73.5)	68.3(63.2–73.0)	0.983	68.6(61.8–74.8)	66.7(63.0–70.3)	0.616
Married (%)	86.2(81.7–89.7)	85.1(81.4–88.1)	0.686	87.9(83.6–91.1)	86.3(82.2–89.5)	0.546	82.3(76.3–87.0)	89.1(86.5–91.3)	0.011
H.hold^a^(%)	07.58(5.03–11.2)	05.8(04.0–08.4)	0.354	10.4(07.3–14.4)	09.6(06.9–13.2)	0.762	13.6(09.4–19.2)	06.2(04.6–08.4)	0.001
Educated (%)	55.8(50.0–61.5)	56.7(52.0–61.3)	0.811	66.4(60.8–71.6)	60.9(55.7–65.9)	0.149	60.1(53.0–66.7)	66.4(62.6–70.0)	0.101
Residence (%)	0.3(0–2.4)	23.6(19.9–27.4)	0.000	0(0)	16.8(13.2–21.1)	0.000	0(0)	23.9(20.8–27.4)	0.000
Distance (mean)	05.2(04.8–05.5)	05.6(05.3–06.0)	0.072	05.5(05.2–05.9)	05.5(05.1–05.9)	0.900	05.1(04.7–05.5)	05.3(05.0–05.6)	0.507
SES^b^^,^^c^(%)									
Poor	34.8(29.5–40.5)	31.5(23.7–36.0)		37.9(32.5–43.5)	29.9(25.3–34.9)		40.4(33.7–47.4)	29.1(25.7–32.8)	
Middle	34.1(28.8–39.8)	30.1(26.0–34.6)		32.8(27.7–38.4)	35.3(30.4–40.4)		29.7(23.7–36.5)	32.9(29.3–36.6)	
Least poor	31.0(25.9–36.6)	38.2(33.8–42.9)	0.132	29.1(24.2–34.6)	34.7(29.9–39.9)	0.086	29.7(23.7–36.5)	37.9(34.2–41.7)	0.010
Sought care (%)	78.2(73.1–82.6)	83.7(80.0–86.9)	0.060	80.2(75.2–84.3)	85.4(81.3–88.7)	0.075	67.1(60.2–73.4)	69.7(66.0–73.1)	0.493
Admitted (%)	44.1(38.4–49.9)	43.4(38.9–48.1)	0.858	24.4(19.9–29.7)	29.9(25.3–34.9)	0.123	21.2(16.0–27.5)	23.5(20.3–26.9)	0.502

^a^H.hold = Heads Household

^b^Social Economic Status

^c^Figures may not add up 100 due to rounding

### Costs of obstetric complications care

Women reporting a complication incurred similar mean costs at baseline and midline. The mean costs appeared high for the women in RBF group at endline. However, Mann-Whitney test showed that the median costs were not significantly different between the women in non-RBF and RBF groups across the surveys **Table 3**.The pooled costs incurred by women who received in-patient care for complications across the surveys are shown in **Table 4**. As resource use may differ by level of health facility, the costs are also shown separately by facility type within each group. Women admitted for in-patient care had slightly higher mean costs at BEmOC than at CEmOC health facilities; this result was fairly consistent for each cost category and between women in RBF and non-RBF groups. Although women admitted for care had high mean costs in the RBF group, there were no significant differences in the median costs incurred by women between the two groups. As a percentage of total costs, non medical costs (e.g. transport, food and accommodation) and productivity losses separately accounted for nearly one half of all costs while medical costs accounted for a much smaller percentage, which is expected since health care services are free in public health facilities in Malawi.

**Table 3:** Summary of costs (US$)^a^ of care in women with self-reported complication, by group and survey year.

	Baseline 2013			Midline 2014			Endline 2015		
	Non-RBF	RBF		Non-RBF	RBF		Non-RBF	RBF	
	N = 227	N = 372	P value^b^	N = 239	N = 300	P value^b^	N = 133	N = 445	P value^b^
Mean	6.69	6.19		5.22	5.59		6.34	7.51	
SD^c^	12.01	12.62		25.15	15.31		18.71	22.99	
Median	1.76	1.02	0.382	0.73	0.73	0.544	0.46	1.05	0.429

^a^Exchange rate mid-year 2015, 1 US$ = 550 Malawi Kwacha (MK)

^b^P values estimated using Mann-Whitney test.

^c^SD Standard deviation

**Table 4:** Summary of household costs for women admitted for complication care, by cost-categories and group.

		Non-RBF				RBF			
		BEmOC	CEmOC	Overall		BEmOC	CEmOC	Overall	
		N = 222	N = 21	N = 243	%	N = 247	N = 201	N = 448	%
Medical costs(US $)^a^					***6*.*9***				***2*.*5***
	Mean	0.91	1.34	0.95		0.58	0.15	0.38	
	SD^b^	3.09	5.21	3.32		2.42	1.5	2.06	
	Median	0	0	0		0	0	0	
Transport & other^c^(US$)^a^					***48*.*7***				***49*.*7***
	Mean	7.16	1.48	6.67		7.09	8.07	7.53	
	SD^b^	23.2	1.93	22.23		13.49	26.05	20.1	
	Median	1.82	0.97	1.82		2.37	1.54	2.21	
Productivity costs(US$)^a^					***44*.*2***				***47*.*7***
	Mean	5.81	8.65	6.05		7.97	6.34	7.24	
	SD^b^	10.44	10.49	10.46		16.11	11.19	14.13	
	Median	3.1	4.63	3.19		3.81	2.61	3.04	
Total costs(US$)^a^^,^^d^		3,083.23	240.97	3,324.20	***100***	3,861.11	2,927.27	6,788.39	***100***
	Mean	**13.89**	**11.47**	**13.68**		**15.63**	**14.56**	**15.15**	
	SD^b^	**30.29**	**11.85**	**29.15**		**21.29**	**31.44**	**26.31**	
	Median	**7.73**	**7.44**	**7.71**		**8.50**	**6.40**	**7.34**	

^a^Exchange rate mid-year 2015,1 US$ = 550 MK

^b^SD Standard deviation

^c^Other costs include food and accommodation

^d^No statistical differences in medians for total costs between RBFand non-RBFgroups,P = 0.729. Mann-Whitney test.

The expected mean costs for obstetric complications were not significantly different between women in non-RBF and RBF groups. This was the case both for all women seeking care with reported complications (the full model) and when only women who ended up admitted for in-patient care were included (restricted model)**Table 5**. The full model showed significant negative associations between costs and parity, women heading households, registration for incentives and the middle poor, meaning that women with these attributes had lower expected mean costs of care. The full model also showed significant evidence of positive association between cost and increasing number of in facility days and, as might be expected, between costs and in-patient care, the proxy for complication severity. The expected mean costs increased by 7.8% (95% CI:6.1–9.6) for each additional day in a facility and was 945.4% (95%CI: 843.7–1,058.8) greater for women who received in-patient care. In the restricted model associations were similar, except that heading household and being middle poor status were no longer significantly negatively associated with costs, while residence in urban areas was.

**Table 5:** Effects of RBF on household costs,adjusted for covariates.

	Full costs model: N = 1,716			Restricted costs model: N = 691		
	*Coef*^a^	*95%CI*^b^		*Coef*^a^	*95%CI*^b^	
Year1*Intervention	-0.047	-0.414	0.319	-0.081	-0.718	0.68
Year2*Intervention	0.27	-0.221	0.762	0.431	-0.358	1.221
Age	0.001	-0.004	0.007	0.014	-0.008	0.036
Parity	-0.073	-0.127	-0.019	-0.206	-0.218	-0.194
Married	-0.085	-0.312	0.14	-0.129	-0.314	0.055
Heads Household	-0.106	-0.172	-0.041	-0.327	-0.672	0.017
Educated	0.049	-0.129	0.227	0.181	-0.071	0.434
Residence	-0.263	-0.583	0.056	-0.467	-0.882	-0.051
Distance	-0.001	-0.017	0.013	-0.003	-0.01	0.002
Facility	0.066	-0.152	0.286	0.081	-0.119	0.156
Registered	-0.203	-0.318	-0.087	-0.501	-0.818	-0.183
Days	0.075	0.059	0.092	0.069	0.061	0.078
In-patient	2.34	2.244	2.45			
SES						
Middle	-0.063	-0.112	-0.015	-0.22	-0.59	0.148
Least poor	-0.006	-0.194	0.182	-0.109	-0.815	0.596
*Constant*	6.22	5.805	6.635	8.178	7.708	8.648

^a^Coefficient

^b^95% Confidence interval

* Denotes interaction

### Time taken to present for obstetric complications care

At both baseline and endline, women with reported complications in the RBF group on average took more days before presenting for care than they did at endline. At baseline and midline, the median duration to seek care was similar for women with reported complications between the two groups but women in RBF group took significantly less median duration (p = 0.025) before presenting for care at endline **Table 6**.

**Table 6:** Time to seek care in days among women with self-reported obstetric complications, by group and survey year.

	Baseline 2013			Midline 2014			Endline 2015		
	Non-RBF	RBF		Non-RBF	RBF		Non-RBF	RBF	
	N = 227	N = 372	P value*	N = 239	N = 300	P value*	N = 133	N = 445	P value*
Mean	2.2	3.1		4.5	4.6		5.9	5.5	
SD^a^	5.3	9.1		9.0	7.5		6.1	8.4	
Median	0	0	0.294	1.0	1.9	0.122	5.0	2.0	0.025

*P values estimated using Mann-Whitney test.

^a^SD Standard Deviation

The expected mean time taken to present for obstetric complications were significantly lower for women in RBF compared to women in the non-RBF group. This was the case both for all women seeking care for reported complications (the full model) and when only women who ended up as in-patients were included (restricted model). In both models, the estimated effects were much stronger in the second year of RBF implementation **Table 7**. In the full model, women in RBF group in year 1 took 27.3% (95% CI:28.4–25.9) less while in Year 2 they took 34.2% (95%CI: 37.8–30.4) less time to care compared to women in non-RBF group. As for attributes influencing time to seek care, there were subtle differences in significance patterns across the two models. The full time model showed significant positive association between increasing age, being married and registration for incentives and time whereas parity, education and in-patient care(disease severity) were significantly negatively associated with time. Women who ended up admitted for in-patient care took 63.7%(95%CI: 73.9–49.5)less time to present for care than women with reported complications but who were not admitted for care, and the effect was statistically significant. As the decision to admit women who presented for care was made at facilities and clinical assessments for inpatient care are largely based on complication severity, this finding means that women who experienced severe complications in the RBF group on average took much less time to present for care.

In the restricted time model, being married was the only attribute significantly positively associated with time while age, education, distance and middle poor were significantly negatively associated with time. The important negative association between distance and time may seem surprising and unexpected. However, if distance is sufficiently long, women may be forced to pay for motorized transport in any case which could be quicker than un-motorized short-travel.

**Table 7:** Effects of RBF on time to seek care (days) for obstetric complications, adjusted for covariates.

	Full Time Model: N = 1, 716			Restricted Time Model: N = 691		
	*Coef*.^a^	*95% CI*^b^		*Coef*.^a^	*95% CI*^b^	
Year1*Intervention	-.318	-.335	-.300	-.458	-.568	-.348
Year2*Intervention	-.419	-.476	-.363	-.835	-1.084	-.585
Age	.010	.004	.015	-.016	-.024	-.009
Parity	-.083	-.102	-.064	-.293	-.341	-.245
Married	.135	.038	.233	.697	.468	.926
Heads Household	.101	-.057	.260	.325	-.196	.848
Educated	-.200	-.295	-.104	-.602	-.829	-.374
Residence	.170	-.030	.370	-.392	-.886	.102
Distance	.013	-.014	.040	-.016	-.028	-.005
Facility	-.057	-.224	.109	.391	-.150	.933
Registered	.166	.101	.231	.397	-.136	.931
Inpatient	-1.014	-1.344	-.684			
SES						
Middle	.044	-.142	.231	-.289	-.457	-.121
Least poor	.065	-.004	.136	-.140	-.497	.215
Constant	.906	.770	1.041	.135	.020	.250

^a^Coefficient

^b^95% Confidence interval

* Denotes interaction

## Discussion

This study makes a unique contribution to the literature since it is the first to describe costs and time to seek care for obstetric complications within the context of RBF. Results indicate that RBF substantially reduced time to seek care for women experiencing an obstetric complication, while RBF did not produce any substantial effect on related overall household costs.

### Costs of obstetric complications care

Our findings indicate that in settings like Malawi which do not impose formal user fees, it may be difficult for RBF to produce a significant effect on household costs associated with seeking care, when both direct and indirect costs are considered at once. Nevertheless, the observation that indirect costs were substantially lower for households that benefited from CCT suggests that RBF has the potential to reduce overall burden on the households. Unfortunately, the data at our disposal makes it impossible to assess the overall social health protection effect of this observed reduction in indirect costs. Our findings are consistent with findings by McIntyre D et al[38], indicating that other direct costs(e.g. transports, food, accommodation) and indirect costs represent a substantially higher burden for households than medical costs alone. In turn, this suggests the importance of intervention that can lower these costs, even when these interventions are unable to affect direct medical costs. Our findings on reduced expected mean costs and reduced informal caregiver engagement among women receiving CCT suggest that cash receipts can substitute for informal caregivers’ time or support. Therefore, among beneficiaries, fewer informal caregivers per case allows households to minimise productivity loses sufficiently to lower overall household costs. Qualitative inquiries can further aid understanding on how this plays out in practice.

### Time to seek obstetric complication care

The mean time to care for obstetric complications was significantly lower for women subjected to RBF intervention. This effect was stronger in the longer rather than shorter term. The finding that financial reimbursements are associated with reduced delays in seeking emergency care is similar to that published by Nahar S et al [20] even though their work is not within a formal RBF context. There are a number of possible explanations for the observed reduction in time to seek care in our setting. Supply side improvements in quality of care may have occurred in intervention facilities, inclining household decision making towards early care seeking. The promise of transport refunds may have emboldened beneficiaries to increase fiscal expenditure thresholds, allowing them to use relatively expensive, but quick modes of transport to get to facilities. Alternatively, the prompt care seeking noted in intervention areas could have been part of a response to broader health education/promotion efforts in the areas informing women about obstetric dangers signs and encouraging them to seek formal care early, or a more functional referral system may have existed in intervention areas. We examine each of these plausible explanations in turn.

First, there is consensus that quality health services attract women to formal care[39–41]. Project documents provided evidence of attendant improvements in structural quality for intervention facilities as a result of equipment and other supplies provided as part of RBF4MNC to strengthen facility capacities. Because the majority of women in the study areas at least attend one antenatal care visit[27], it is probable that engagement with better antenatal care services during preceding visit(s) may have “primed” the women’s perceptions regarding improved quality of care at intervention facilities, leading to subsequent prompt care seeking in times of potential obstetric emergencies. Second, guarantees of transport refunds could have empowered potential beneficiaries to use their fiscal resources to pay for motorized modes of transport. Alternatively, guarantees of cash refunds could have reduced perceived financial constraints allowing household to take immediate decisions to seek emergency obstetric care. Regarding the former, our data do not support this assertion as the percentage of households that used any motorized form of transport (e.g. cars) did not significantly differ between intervention and control areas. Women registered for incentives had significantly higher expected mean time to care, which does not support our premise that perceptions of fiscal empowerment may have promoted prompt decision making. Third, health education is an integral part of RH services provided to antenatal women. Centrally planned and coordinated, standardized reproductive health education is evenly provided across all facilities in a district. Although local non-governmental organizations are increasingly taking part and supporting DHMT in health promotion activities in the study districts, we have no evidence that intervention areas received any special intensive health promotion activities. In fact, our data shows that care seeking patterns were not different between intervention and control groups **(Table 1).** Fourth, even though referral systems were not explicitly incentivised, there is a possibility that (presumably) motivated health providers in intervention BEmOC facilities could have coordinated better with CEmOC facilities to arrange transport for the women with complications, for those referred from BEmOC to CEmOC facilities. There is evidence that differential transport arrangements existed between intervention and control facilities, but this was in favour of referrals from control BEmOCs. Ruling out these alternatives, we conclude that the significantly reduced time to care observed in intervention areas most likely resulted from prompt decision making at household level due to perceptions of facility quality improvement, while community level delays appear to be less important.

From a policy perspective, it is important that women with higher risk profiles for obstetric complications (e.g. high parity or the poor) present for curative care early. It is therefore worth exploring how responsive women with different risk attributes were to RBF. We found that high parity, education, increasing distance and medium poor status were associated with significantly lesser expected mean time to care. The experience that comes with more births (high parity) and information associated with high education allows women to make better decisions as might be expected. That the medium poor respond faster than the poor reiterates the usual disadvantage faced by the poor.

It is a fair question to ask what influence different components of RBF4MNH had on primary study outcomes. The observed short term effects give an estimate of what to expect if only supply-side incentives were in place; a significantly reduced mean time to care but no substantial change in overall household costs. Unfortunately, estimating with certainty any additional effects accruing from a combination of supply and demand-side incentives is not possible in our study given the low coverage (25%) for demand-side incentives. Because this would be valuable information for policy makers, studies based on optimally designed and implemented RBF programs that allow for such detailed evaluations are needed. Information on relative effectiveness of RBF components will provide more policy options: enabling better configuration of financial incentive structures to align with local health priorities and health systems capacities.

Our study can not explain how RBF influence power dynamics in the home in view of the fact that in many societies, it’s the men who make key decision related to health choices[42]. Neither can it shed light on how actually the women/ households mobilized resources to finance transport during emergencies. These are potential qualitative research question for future studies. Finally, for countries like Malawi where donors provide a large share of healthcare budget[43], it is important to consider sustainability and cost-effectiveness of RBF in developing countries[44, 45].

### Limitations

This study has some limitations. First, women with complications self-select for obstetric care. The women who did not seek care for reported complications could thus bias the results. Since the intervention did not produce significant effects on overall service use between the two groups across the survey years, we do not anticipate a large selection bias. Second, as the information was collected retrospectively, recall bias resulting in time-varying deferential reporting of study outcomes may have affected the results. As four week recall is often used in cost studies, we compared mean costs/ time estimates reported within 4 weeks of termination of pregnancy with those reported after 4 weeks as validation checks and to assess size of bias, if any. We made these comparisons between groups, before and after the implementation of intervention. The results (available upon request) demonstrate no influence of recall bias on time estimates but suggest recall bias may have affected cost estimates in the post intervention period. We thus argue that our results should be read with these limitations in mind.

## Conclusions

The most important finding of this study is the significant reduction in the expected mean time taken before presenting for obstetric complication care by recipients of RBF. This occurred despite the lack of a substantial change in overall household costs. This result is probably a manifestation of the RBF induced quality improvements which encourage immediately care seeking when women are faced with potential obstetric complications. Our results suggest RBF may contribute to prompt emergency care seeking and thus ultimately reduce maternal morbidity and mortality in beneficiary populations.

## Supporting information

S1 Data (XLSX)
